# Supplementary material for: Influence of the ATP-dependent DNA ligase, Lig E, on Neisseria gonorrhoeae microcolony and biofilm formation
Source: Biofilm. 2025 Jun 5;10:100292. doi: 10.1016/j.bioflm.2025.100292 (PMC12221376; doi:10.1016/j.bioflm.2025.100292)
Supplement: Multimedia component 1 [file mmc1.docx]

# **Supplementary Information:** Influence of the ATP-dependent DNA ligase, Lig E, on *Neisseria gonorrhoeae* microcolony and biofilm formation

Jolyn Pan^1^, Abdullah Albarrak^2^, Joanna Hicks^3^, David Williams^2^, Adele Williamson^1^

*^1^School of Science, University of Waikato, Hamilton, New Zealand*

*^2^School of Dentistry, Cardiff University, Wales, United Kingdom*

*^3^School of Health, University of Waikato, Hamilton, New Zealand*


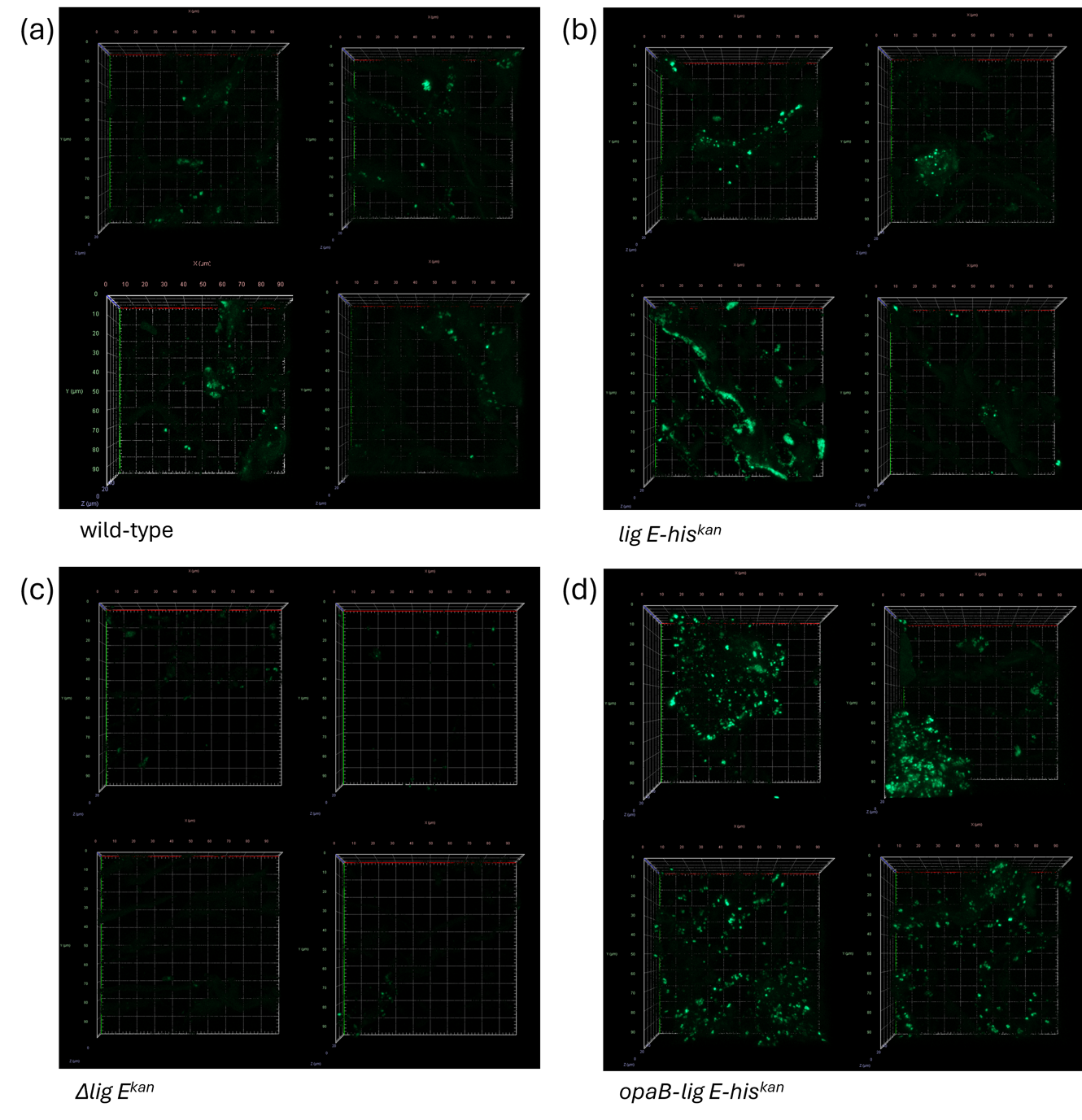


Figure S1. Additional CLSM z-stack images of representative fields-of-view of *N. gonorrhoeae* pEG2 (expressing sfGFP) biofilm formation and adhesion on polycarbonate coupons in CDC Biofilm Reactors® (x40 magnification, em:480 nm, exc:505 nm).


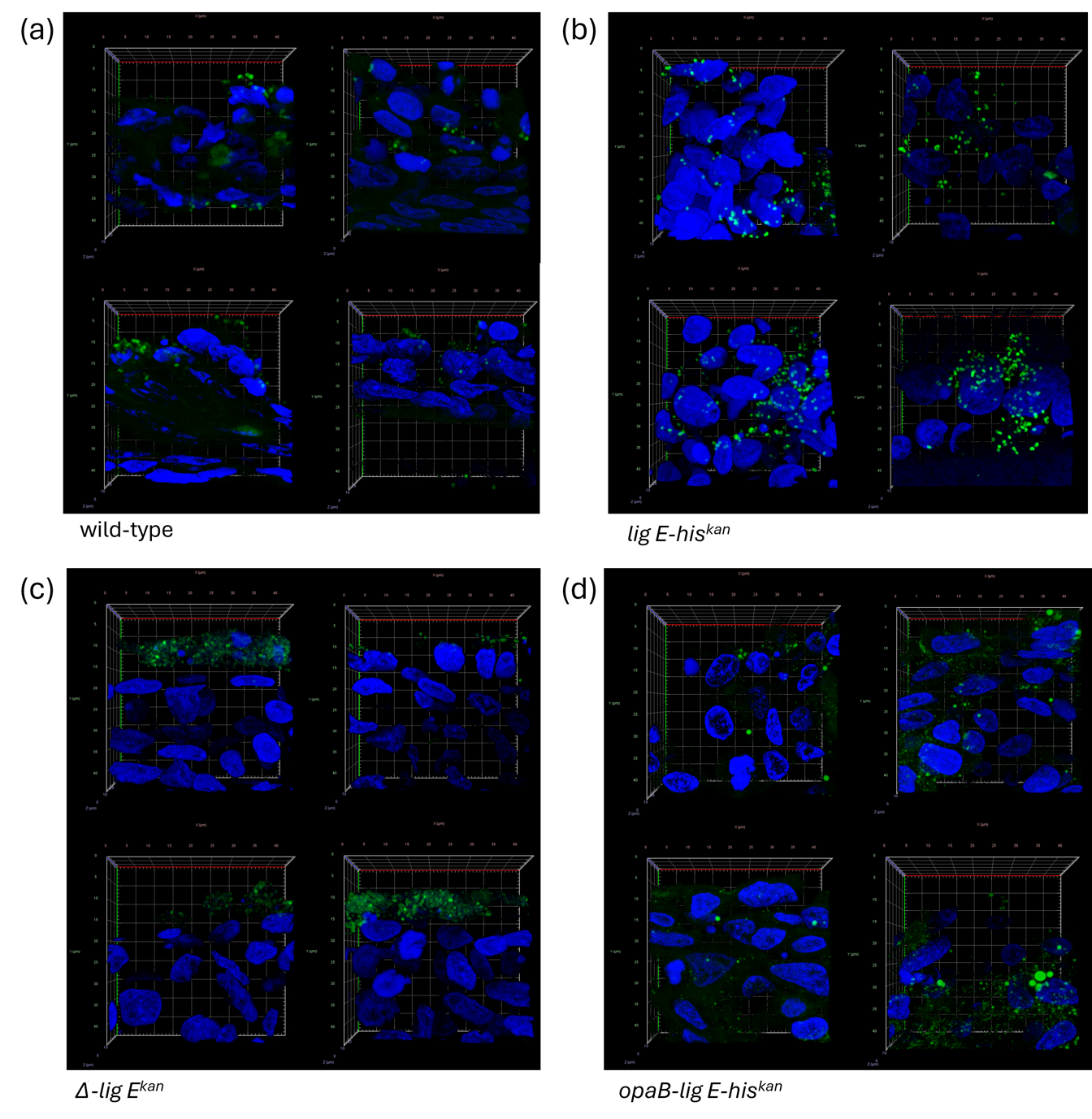


Figure S2. Additional CLSM z-stack images (x63 objective magnification, zoom setting 3) of representative fields-of-view of the infection and invasion of SkinEthic^TM^ HVE cells by *N. gonorrhoeae*. *N. gonorrhoeae* pEG2 (expressing sfGFP) is shown in green (exc: 488 nm, em: 519 nm) and the nuclei of the rHVE cells in blue (exc: 405 nm, em: 449 nm).


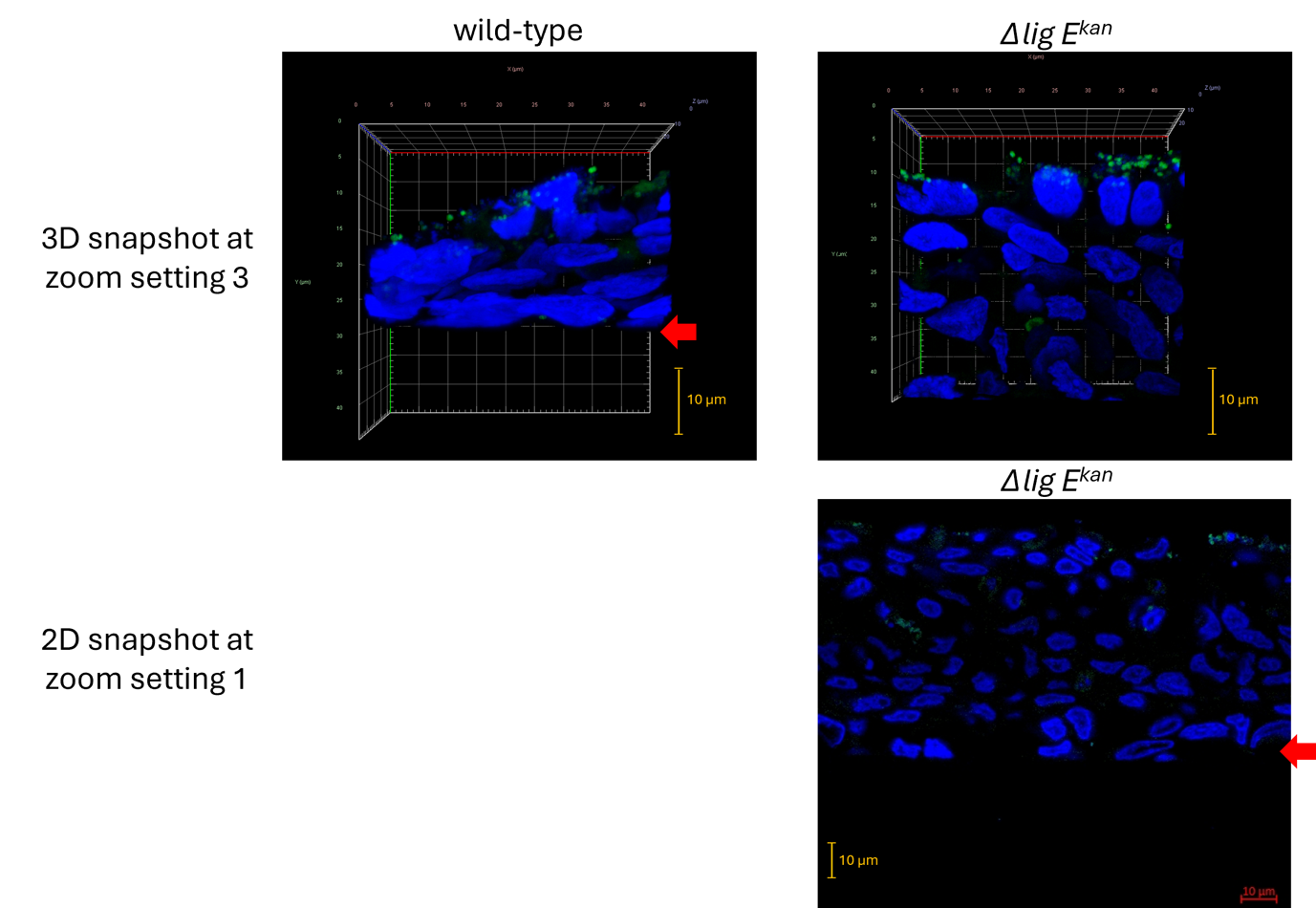


Figure S3. CLSM images (x63 objective magnification) of SkinEthic^TM^ HVE cells (blue, exc: 405 nm, em: 449 nm) infected with *N. gonorrhoeae* pEG2 (green, exc: 488 nm, em: 519 nm) showing the extent of tissue damage. The top row shows the 3-D snapshot of *wt* and *Δlig E^kan^* *N. gonorrhoeae* at zoom setting 3 (final zoom setting used), showing the depth and length of the remaining intact tissue, with the tissue membrane visible for *wt* at that setting (red arrow). The bottom row shows a 2-D snapshot of the same *Δlig E^kan^* infected tissue slide at zoom setting 1 which finally shows the membrane of the tissue (red arrow).


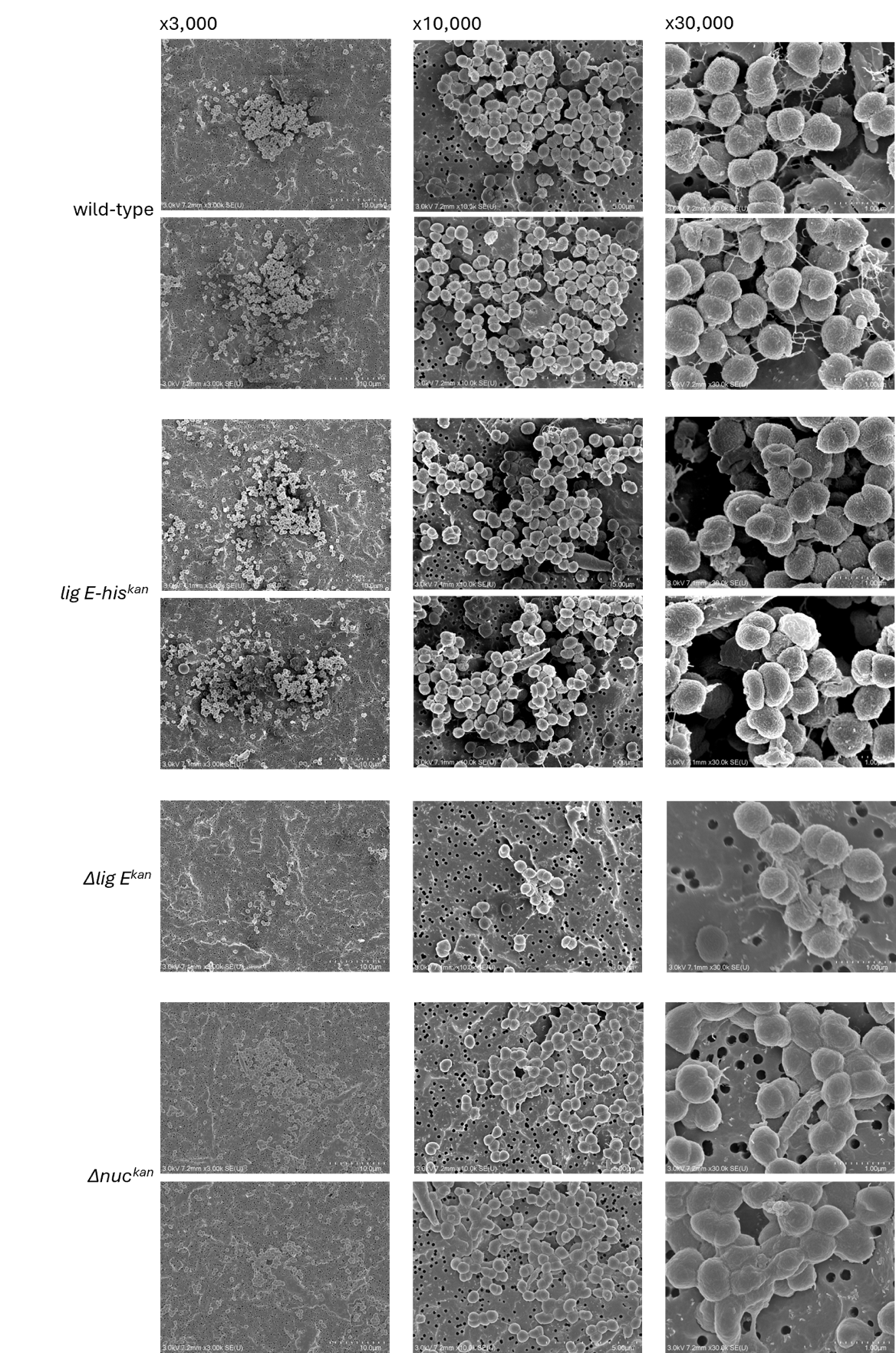


Figure S4. Additional supporting SEM images of *N. gonorrhoeae* microcolonies formed on 0.2 µm pore size filter paper during the exponential phase of growth (9 hours). Note, only one other field-of-view is shown for *Δlig E^kan^* due to the lack of microcolonies formed by this mutant.

Table S1. Raw data and measurement of the ratio of *N. gonorrhoeae* infection relative to rHVE depth presented in Figure 4C.

| **Wild-type** | | | | | | | | |
| --- | --- | --- | --- | --- | --- | --- | --- | --- |
|  |  | Green channel (exc: 488 nm, em: 519 nm) - N. gonorrhoeae peg2 | | | Blue channel (exc: 405 nm, em: 449 nm) - rHVE nuclei | | |  |
| Replicate | Field-of-view | Highest point on y-axis (µm) | Lowest point on y-axis (µm) | Distance/depth on the y-axis (µm) | Highest point on y-axis (µm) | Lowest point on y-axis (µm) | Distance/depth on the y-axis (µm) | **Ratio of N. gonorrhoeae depth over rHVE nuclei depth** |
| 1A | 1 | 1 | 30 | 29 | 4 | 30 | 26 | **1.115385** |
|  | 2 | 0 | 41 | 41 | 4 | 34 | 30 | **1.366667** |
|  | 3 | 2 | 43 | 41 | 6 | 43 | 37 | **1.108108** |
|  | 4 | 0 | 35 | 35 | 3.5 | 45 | 41.5 | **0.843373** |
|  | 5 | 15 | 17 | 2 | 15 | 45 | 30 | **0.066667** |
| 1B | 1 | 0 | 35 | 35 | 1 | 45 | 44 | **0.795455** |
|  | 2 | 0 | 45 | 45 | 4 | 45 | 41 | **1.097561** |
|  | 3 | 1 | 33 | 32 | 4 | 45 | 41 | **0.780488** |
|  | 4 | 16 | 45 | 29 | 20 | 45 | 25 | **1.16** |
|  | 5 | 0.5 | 33 | 32.5 | 5 | 38.5 | 33.5 | **0.970149** |
| 2A | 1 | 13 | 18 | 5 | 15 | 26.5 | 11.5 | **0.434783** |
|  | 2 | 11 | 23 | 12 | 15 | 30 | 15 | **0.8** |
|  | 3 | 10 | 16 | 6 | 11 | 35 | 24 | **0.25** |
|  | 4 | 10 | 29 | 19 | 10 | 40 | 30 | **0.633333** |
|  | 5 | 8 | 37 | 29 | 15 | 42 | 27 | **1.074074** |
| 2B | 1 | 5 | 45 | 40 | 7 | 23 | 16 | **2.5** |
|  | 2 | 15 | 45 | 30 | 17 | 37 | 20 | **1.5** |
|  | 3 | 5 | 45 | 40 | 5 | 29 | 24 | **1.666667** |
|  | 4 | 7 | 43 | 36 | 7 | 30 | 23 | **1.565217** |
|  | 5 | 16 | 33 | 17 | 14 | 31 | 17 | **1** |
| 3A | 1 | 8 | 34 | 26 | 7 | 38 | 31 | **0.83871** |
|  | 2 | 9 | 33 | 24 | 10 | 44 | 34 | **0.705882** |
|  | 3 | 3 | 40 | 37 | 5 | 37 | 32 | **1.15625** |
|  | 4 | 0 | 19 | 19 | 2 | 36 | 34 | **0.558824** |
|  | 6 | 0 | 45 | 45 | 0 | 45 | 45 | **1** |
| 3B | 1 | 0 | 28 | 28 | 0 | 45 | 45 | **0.622222** |
|  | 2 | 4 | 43 | 39 | 3 | 24 | 21 | **1.857143** |
|  | 3 | 0 | 19 | 19 | 1 | 32 | 31 | **0.612903** |
|  | 4 | 5 | 25 | 20 | 5.5 | 35 | 29.5 | **0.677966** |
|  | 5 | 3 | 35 | 32 | 3 | 35 | 32 | **1** |
|  |  |  | **Average** | **28.15** |  |  | **29.7** | **0.991928** |
|  |  |  | **stdev** | **11.77311** |  |  | **9.107216** | **0.490162** |
|  |  |  |  |  |  |  |  |  |
|  |  |  |  |  |  |  |  |  |
| ***lig E-his^kan^*** | | | | | | | | |
|  |  | Green channel (exc: 488 nm, em: 519 nm) - N. gonorrhoeae peg2 | | | Blue channel (exc: 405 nm, em: 449 nm) - rHVE nuclei | | |  |
| Replicate | Field-of-view | Highest point on y-axis (µm) | Lowest point on y-axis (µm) | Distance/depth on the y-axis (µm) | Highest point on y-axis (µm) | Lowest point on y-axis (µm) | Distance/depth on the y-axis (µm) | **Ratio of N. gonorrhoeae depth over rHVE nuclei depth** |
| 1A | 1 | 3 | 18 | 15 | 7 | 45 | 38 | **0.394737** |
|  | 2 | 1 | 16 | 15 | 9 | 45 | 36 | **0.416667** |
|  | 3 | 3 | 32 | 29 | 4 | 45 | 41 | **0.707317** |
|  | 4 | 0 | 26 | 26 | 0 | 45 | 45 | **0.577778** |
|  | 5 | 6 | 39 | 33 | 0 | 45 | 45 | **0.733333** |
| 1B | 1 | 5 | 45 | 40 | 19 | 45 | 26 | **1.538462** |
|  | 2 | 12 | 27 | 15 | 6.5 | 45 | 38.5 | **0.38961** |
|  | 3 | 2 | 45 | 43 | 9 | 45 | 36 | **1.194444** |
|  | 4 | 8 | 45 | 37 | 9 | 45 | 36 | **1.027778** |
|  | 5 | 1.5 | 45 | 43.5 | 0 | 45 | 45 | **0.966667** |
| 2A | 1 | 0 | 40 | 40 | 1 | 45 | 44 | **0.909091** |
|  | 2 | 2 | 45 | 43 | 0 | 45 | 45 | **0.955556** |
|  | 3 | 4 | 45 | 41 | 10.5 | 38.5 | 28 | **1.464286** |
|  | 4 | 1 | 45 | 44 | 3 | 45 | 42 | **1.047619** |
|  | 5 | 1.5 | 45 | 43.5 | 13 | 45 | 32 | **1.359375** |
| 2B | 1 | 0 | 45 | 45 | 0 | 45 | 45 | **1** |
|  | 2 | 1 | 45 | 44 | 11.5 | 45 | 33.5 | **1.313433** |
|  | 3 | 1 | 33 | 32 | 1.5 | 45 | 43.5 | **0.735632** |
|  | 4 | 0 | 45 | 45 | 1.5 | 45 | 43.5 | **1.034483** |
|  | 5 | 1.5 | 44 | 42.5 | 12 | 45 | 33 | **1.287879** |
| 3A | 1 | 7 | 40 | 33 | 11 | 45 | 34 | **0.970588** |
|  | 2 | 7 | 37 | 30 | 10 | 45 | 35 | **0.857143** |
|  | 3 | 7 | 45 | 38 | 10 | 45 | 35 | **1.085714** |
|  | 4 | 3 | 31 | 28 | 10 | 45 | 35 | **0.8** |
|  | 5 | 0 | 35 | 35 | 5 | 45 | 40 | **0.875** |
| 3B | 1 | 0 | 45 | 45 | 0 | 45 | 45 | **1** |
|  | 2 | 2 | 45 | 43 | 2 | 45 | 43 | **1** |
|  | 3 | 0 | 35 | 35 | 1 | 45 | 44 | **0.795455** |
|  | 4 | 0 | 45 | 45 | 0 | 45 | 45 | **1** |
|  | 5 | 0 | 45 | 45 | 1 | 31 | 30 | **1.5** |
|  |  |  | **Average** | **36.45** |  |  | **38.73333** | **0.964602** |
|  |  |  | **stdev** | **9.282306** |  |  | **5.747163** | **0.304664** |
|  |  |  |  |  |  |  |  |  |
| ***Δlig E^kan^*** | | | | | | | | |
|  |  | Green channel (exc: 488 nm, em: 519 nm) - N. gonorrhoeae peg2 | | | Blue channel (exc: 405 nm, em: 449 nm) - rHVE nuclei | | |  |
| Replicate | Field-of-view | Highest point on y-axis (µm) | Lowest point on y-axis (µm) | Distance/depth on the y-axis (µm) | Highest point on y-axis (µm) | Lowest point on y-axis (µm) | Distance/depth on the y-axis (µm) | **Ratio of N. gonorrhoeae depth over rHVE nuclei depth** |
| 1A | 1 | 4 | 17 | 13 | 5 | 45 | 40 | **0.325** |
|  | 2 | 0 | 10 | 10 | 0 | 45 | 45 | **0.222222** |
|  | 3 | 3 | 13 | 10 | 3 | 45 | 42 | **0.238095** |
|  | 4 | 4 | 10 | 6 | 6 | 45 | 39 | **0.153846** |
|  | 5 | 3 | 8 | 5 | 3 | 45 | 42 | **0.119048** |
| 1B | 1 | 6 | 16 | 10 | 5 | 45 | 40 | **0.25** |
|  | 2 | 2 | 15 | 13 | 5 | 45 | 40 | **0.325** |
|  | 3 | 4 | 18 | 14 | 6 | 45 | 39 | **0.358974** |
|  | 4 | 2 | 9 | 7 | 5 | 45 | 40 | **0.175** |
|  | 5 | 3 | 16 | 13 | 12.5 | 45 | 32.5 | **0.4** |
| 2A | 1 | 10 | 13 | 3 | 11 | 45 | 34 | **0.088235** |
|  | 2 | 0 | 0 | 0 | 1 | 45 | 44 | **0** |
|  | 3 | 12 | 14 | 2 | 10 | 45 | 35 | **0.057143** |
|  | 4 | 9 | 23 | 14 | 8 | 45 | 37 | **0.378378** |
|  | 5 | 10 | 21 | 11 | 9 | 45 | 36 | **0.305556** |
| 2B | 1 | 11 | 13 | 2 | 9 | 45 | 36 | **0.055556** |
|  | 2 | 3 | 24 | 21 | 2 | 45 | 43 | **0.488372** |
|  | 3 | 1 | 16 | 15 | 1 | 45 | 44 | **0.340909** |
|  | 4 | 6 | 24 | 18 | 3 | 45 | 42 | **0.428571** |
|  | 5 | 6 | 14 | 8 | 3 | 45 | 42 | **0.190476** |
| 3A | 1 | 0 | 45 | 45 | 6 | 45 | 39 | **1.153846** |
|  | 2 | 1 | 13 | 12 | 0 | 45 | 45 | **0.266667** |
|  | 3 | 5 | 45 | 40 | 4 | 45 | 41 | **0.97561** |
|  | 4 | 6 | 45 | 39 | 5 | 45 | 40 | **0.975** |
|  | 5 | 6 | 45 | 39 | 3 | 45 | 42 | **0.928571** |
| 3B | 1 | 10 | 45 | 35 | 11 | 45 | 34 | **1.029412** |
|  | 2 | 3 | 45 | 42 | 8 | 45 | 37 | **1.135135** |
|  | 3 | 0 | 45 | 45 | 0 | 45 | 45 | **1** |
|  | 4 | 0 | 45 | 45 | 0 | 45 | 45 | **1** |
|  | 5 | 15 | 45 | 30 | 8 | 45 | 37 | **0.810811** |
|  |  |  | **Average** | **18.9** |  |  | **39.91667** | **0.472514** |
|  |  |  | **stdev** | **15.01115** |  |  | **3.605751** | **0.373113** |
|  |  |  |  |  |  |  |  |  |
| ***opaB-lig E^kan^*** | | | | | | | | |
|  |  | Green channel (exc: 488 nm, em: 519 nm) - N. gonorrhoeae peg2 | | | Blue channel (exc: 405 nm, em: 449 nm) - rHVE nuclei | | |  |
| Replicate | Field-of-view | Highest point on y-axis (µm) | Lowest point on y-axis (µm) | Distance/depth on the y-axis (µm) | Highest point on y-axis (µm) | Lowest point on y-axis (µm) | Distance/depth on the y-axis (µm) | **Ratio of N. gonorrhoeae depth over rHVE nuclei depth** |
| 1A | 1 | 8 | 39 | 31 | 5 | 45 | 40 | **0.775** |
|  | 2 | 0 | 25 | 25 | 2 | 45 | 43 | **0.581395** |
|  | 3 | 13 | 14 | 1 | 1 | 45 | 44 | **0.022727** |
|  | 4 | 13 | 26 | 13 | 5 | 45 | 40 | **0.325** |
|  | 5 | 6 | 26 | 20 | 3 | 45 | 42 | **0.47619** |
| 1B | 1 | 4 | 15 | 11 | 0 | 45 | 45 | **0.244444** |
|  | 2 | 4 | 39 | 35 | 1 | 45 | 44 | **0.795455** |
|  | 3 | 10 | 45 | 35 | 3.5 | 45 | 41.5 | **0.843373** |
|  | 4 | 11 | 43 | 32 | 4 | 45 | 41 | **0.780488** |
|  | 5 | 10 | 30 | 20 | 5 | 45 | 40 | **0.5** |
|  | 6 | 20 | 45 | 25 | 10 | 45 | 35 | **0.714286** |
|  | 7 | 0 | 45 | 45 | 1 | 45 | 44 | **1.022727** |
| 2A | 1 | 16 | 17 | 1 | 12 | 25 | 13 | **0.076923** |
|  | 2 | 0 | 0 | 0 | 17 | 24 | 7 | **0** |
|  | 3 | 0 | 0 | 0 | 17 | 26 | 9 | **0** |
|  | 4 | 0 | 0 | 0 | 7 | 16 | 9 | **0** |
|  | 5 | 0 | 7 | 7 | 1 | 15 | 14 | **0.5** |
| 3A | 1 | 8 | 11 | 3 | 3 | 45 | 42 | **0.071429** |
|  | 2 | 10 | 13 | 3 | 8 | 45 | 37 | **0.081081** |
|  | 3 | 7 | 12 | 5 | 8 | 45 | 37 | **0.135135** |
|  | 4 | 9 | 14 | 5 | 4 | 45 | 41 | **0.121951** |
|  | 5 | 0 | 14 | 14 | 3 | 45 | 42 | **0.333333** |
| 3B | 1 | 9 | 27 | 18 | 7 | 45 | 38 | **0.473684** |
|  | 2 | 26 | 35 | 9 | 1 | 45 | 44 | **0.204545** |
|  | 3 | 2 | 9 | 7 | 1 | 45 | 44 | **0.159091** |
|  | 4 | 10 | 36 | 26 | 3 | 45 | 42 | **0.619048** |
|  | 5 | 19 | 40 | 21 | 3 | 45 | 42 | **0.5** |
|  | 6 | 32 | 33 | 1 | 8 | 45 | 37 | **0.027027** |
|  | 7 | 0 | 0 | 0 | 7 | 45 | 38 | **0** |
|  | 8 | 0 | 0 | 0 | 3 | 45 | 42 | **0** |
|  |  |  | **Average** | **13.76667** |  |  | **35.91667** | **0.346144** |
|  |  |  | **stdev** | **13.13244** |  |  | **11.91644** | **0.312581** |

Table S2. Raw data of absorbance at 490 nm corrected for background absorbance at 680 nm for LDH quantification in Figure 5.

|  | **wild-type** | ***lig E-his^kan^*** | ***Δlig E^kan^*** | ***opaB-lig E^kan^*** | **uninfected tissue** |
| --- | --- | --- | --- | --- | --- |
| Biological replicate A | 0.472 | 0.199 | 0.065 | 0.155 | **0.104** |
|  | 0.353 | 0.188 | 0.077 | 0.161 | **0.087** |
|  | 0.383 | 0.172 | 0.071 | 0.141 | **0.092** |
| Biological replicate B | 0.433 | 0.362 | 0.037 | 0.250 | **0.061** |
|  | 0.329 | 0.360 | 0.025 | 0.268 | **0.067** |
|  | 0.307 | 0.265 | 0.012 | 0.258 | **0.071** |
| Biological replicate C | 0.391 | 0.549 | 0.086 | 0.125 | **0.089** |
|  | 0.302 | 0.458 | 0.074 | 0.125 | **0.102** |
|  | 0.260 | 0.370 | 0.067 | 0.111 | **0.091** |
| **Average** | **0.359** | **0.325** | **0.0575** | **0.1775** | **0.085** |

Table S3. Data of corrected absorbance from Table S2 normalised to the average value of uninfected tissue.

|  | **wild-type** | ***lig E-his^kan^*** | ***Δlig E^kan^*** | ***opaB-lig E^kan^*** | **uninfected tissue** |
| --- | --- | --- | --- | --- | --- |
| Biological replicate A | 5.560209 | 2.344241 | 0.765707 | 1.825916 | 1.225131 |
|  | 4.158377 | 2.21466 | 0.907068 | 1.896597 | 1.024869 |
|  | 4.51178 | 2.026178 | 0.836387 | 1.660995 | 1.08377 |
| Biological replicate B | 5.100785 | 4.264398 | 0.435864 | 2.945026 | 0.718586 |
|  | 3.875654 | 4.240838 | 0.294503 | 3.157068 | 0.789267 |
|  | 3.616492 | 3.121728 | 0.141361 | 3.039267 | 0.836387 |
| Biological replicate C | 4.606021 | 6.467277 | 1.013089 | 1.472513 | 1.048429 |
|  | 3.557592 | 5.395288 | 0.871728 | 1.472513 | 1.201571 |
|  | 3.062827 | 4.358639 | 0.789267 | 1.307592 | 1.07199 |
| **Average** | **4.2277485556** | **3.8259163333** | **0.6727748889** | **2.0863874444** | **1** |


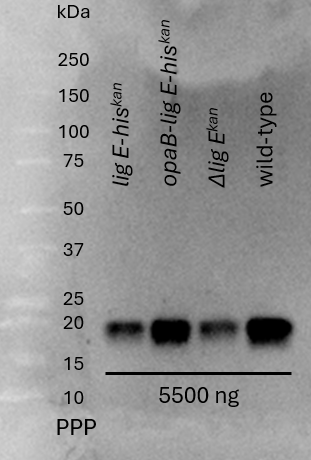


Figure S5. Western blot of *N. gonorrhoeae* peg2 cultures against the EYLLN motif on the conserved N-terminus domain of PilE. Equal amounts of protein were loaded in each well (5500 ng). the predicted size of PilE in *N. gonorrhoeae* MS11 is ~18kDa. The sizes of the ladder (PPP, Protein Precision Plus^TM^ All Blue Prestained Protein Standards (Bio-Rad)) were marked on the membrane after Ponceau staining to determine the sizes of the bands after probing and visualisation).
